# Regeneration of Alveolar Type I and II Cells from Scgb1a1-Expressing Cells following Severe Pulmonary Damage Induced by Bleomycin and Influenza

**DOI:** 10.1371/journal.pone.0048451

**Published:** 2012-10-31

**Authors:** Dahai Zheng, Gino V. Limmon, Lu Yin, Nicola H. N. Leung, Hanry Yu, Vincent T. K. Chow, Jianzhu Chen

**Affiliations:** 1 Interdisciplinary Research Group in Infectious Diseases, Singapore-Massachusetts Institute of Technology Alliance for Research and Technology, Singapore, Singapore; 2 The Koch Institute for Integrative Cancer Research and Department of Biology, Massachusetts Institute of Technology, Cambridge, Massachusetts, United States of America; 3 Institute of Bioengineering and Nanotechnology, Agency for Science, Technology and Research, Singapore; Department of Physiology & Mechanobiology, National University of Singapore, Singapore; 4 Human Genome Laboratory, Department of Microbiology, School of Medicine, National University of Singapore, Singapore; Children's Hospital Los Angeles, United States of America

## Abstract

The lung comprises an extensive surface of epithelia constantly exposed to environmental insults. Maintaining the integrity of the alveolar epithelia is critical for lung function and gaseous exchange. However, following severe pulmonary damage, what progenitor cells give rise to alveolar type I and II cells during the regeneration of alveolar epithelia has not been fully determined. In this study, we have investigated this issue by using transgenic mice in which Scgb1a1-expressing cells and their progeny can be genetically labeled with EGFP. We show that following severe alveolar damage induced either by bleomycin or by infection with influenza virus, the majority of the newly generated alveolar type II cells in the damaged parenchyma were labeled with EGFP. A large proportion of EGFP-expressing type I cells were also observed among the type II cells. These findings strongly suggest that Scgb1a1-expressing cells, most likely Clara cells, are a major cell type that gives rise to alveolar type I and II cells during the regeneration of alveolar epithelia in response to severe pulmonary damage in mice.

## Introduction

It is predicted that lung diseases will rank second in terms of prevalence, mortality, and healthcare costs [1], [2]. The development of lung regenerative medicine is limited by the relative lack of knowledge about stem and progenitor cells and their identity, organization and regulation in the adult lung [2]. The lung comprises many types of epithelial cells that reside in anatomically distinct regions of the trachea, bronchioles and alveoli [3]–[5]. In the mouse, Clara cells are the predominant cell type in the conducting airway, whereas alveolar epithelia are covered by alveolar type I cells (AT1s) interspersed with alveolar type II cells (AT2s). Clara cells are known as important progenitor cells for the maintenance and repair of bronchiolar epithelia [6]–[8]. However, the mechanism of alveolar epithelial regeneration, especially under severe pathologic conditions, is still not clear. Most current knowledge about the regeneration of damaged alveolar epithelia is derived from studies of bleomycin-induced lung injury. Bleomycin is a clinically important anti-cancer agent, but its use is restricted by pulmonary toxicity, including induction of lung fibrosis [9]–[11]. Based on studies of bleomycin-induced alveolar damage, regeneration of alveolar epithelia has long been thought to be mediated by proliferation of existing AT2s and their subsequent differentiation into AT1s [12]–[14]. However, this notion was challenged by a recent lineage tracing study showing that the newly generated AT2s are not derived from the pre-existing AT2s [15]. Based on morphological observations, a previous study also suggested that bronchiolar cells may give rise to AT2s and AT1s during the regeneration of alveolar epithelia [13]. However, this idea has not attracted significant attention due to a lack of definitive evidence.

Recently, various lung stem and progenitor cells have been identified. Bronchioalveolar stem cells (BASCs) express markers of both Clara cells and AT2s, and reside at the broncho-alveolar duct junction (BADJ) [16]. Sca-1 and CD34 are used to isolate these putative BASCs [16], yet the validity of these markers has been questioned by other groups [17], [18]. *In vitro*, BASCs can be induced to differentiate into both Clara cells and AT2s, while their function as stem cells has yet to be evaluated by lineage tracing *in vivo*. A c-kit-positive human lung stem cell was shown to give rise to bronchioles, alveoli and pulmonary vessels after injection into the damaged mouse lung [19]. A mouse lung stem cell was reported to express integrin α6β4, and form clusters of Clara cells and AT2s in kidney capsules when mixed together with fetal lung cells [15]. More recently, it was reported that p63-positive cells in the murine distal lung may contribute to alveolar repair after influenza virus-induced pulmonary injury [20]. However, none of these reported lung stem and progenitor cells has been unequivocally shown to give rise to AT1s and AT2s during the regeneration of alveolar epithelia by genetic lineage tracing *in vivo* [16], [19]–[21].

In our study of Clara cells in lung damage repair, we employed transgenic mice in which Scgb1a1-expressing cells and their progeny can be genetically labeled with EGFP. Here, we show that following bleomycin treatment or influenza virus infection, the majority of the newly generated AT2s in the damaged parenchyma were labeled with EGFP. A large proportion of EGFP-expressing AT1s were also observed among the AT2s. These findings strongly suggest that Scgb1a1-expressing cells, most likely Clara cells, are a major cell type that gives rise to AT1s and AT2s during the regeneration of alveolar epithelia in response to severe pulmonary damage.

## Results

**Figure 1:**
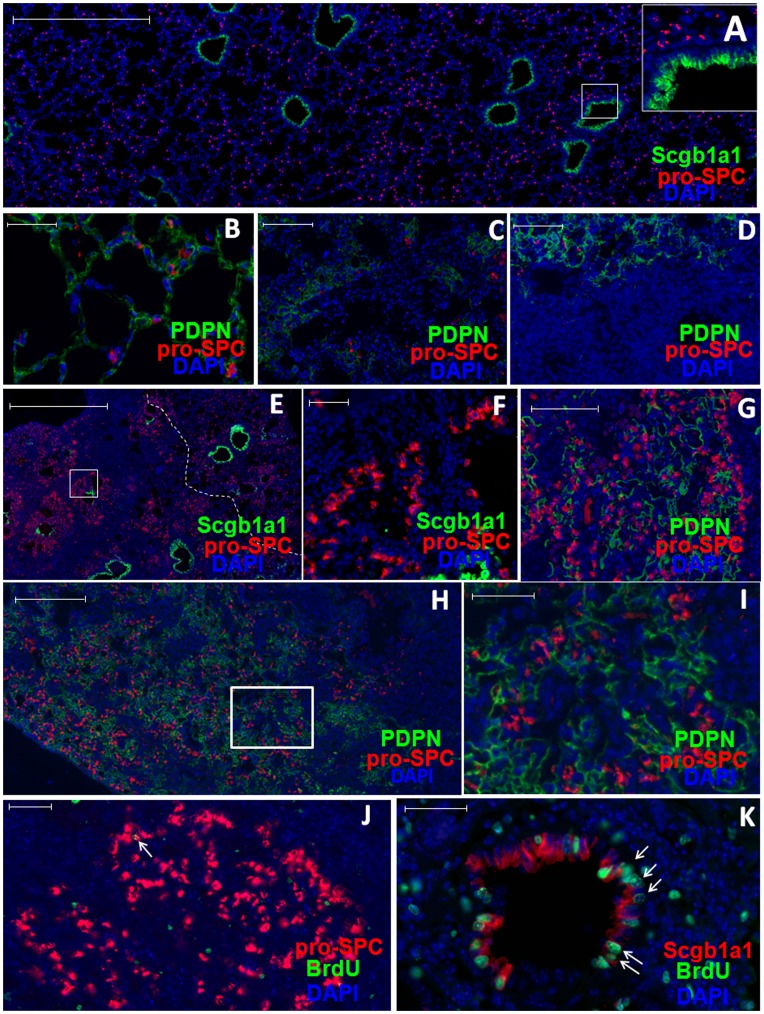
Damage and repair of alveolar epithelia following bleomycin treatment or influenza virus infection. A. Representative lung sections of untreated C57BL/6 (B6) mice displaying Scgb1a1 (green), pro-SPC (red) and DAPI (blue) staining. Higher magnification of the boxed area is shown at the upper-right corner. **B–D.** Representative images of PDPN (green), pro-SPC (red) and DAPI (blue) staining of lung sections of untreated B6 mice (B); or mice at 7 days post-treatment with bleomycin (C); or mice at 11 days post-infection with influenza virus (D). **E&F.** Representative images of Scgb1a1(green), pro-SPC (red) and DAPI (blue) staining of lung sections of B6 mice at 21 days post-bleomycin treatment. Higher magnification of the selected area is shown as (F). White broken line in (E) demarcates the damaged area (left) from the normal area (right) of the lung. **G–I.** Representative images of PDPN (green), pro-SPC (red) and DAPI (blue) staining of lung sections of B6 mice at 21 days post-bleomycin treatment (G), or at 21 days after virus infection (H). Higher magnification of the selected area in (H) is shown as (I). **J–K.** Representative images of pro-SPC (red in J) or Scgb1a1 (red in K), BrdU (green) and DAPI (blue) staining of lung sections of B6 mice at 19 days (J) or 9 days (K) after virus infection, arrows indicate pro-SPC and BrdU double positive (J) or Scgb1a1 and BrdU double positive (K) cells. Note, BrdU-positive cells that are outside of the bronchiole in (K) are negative for pro-SPC. Scale bars: (A) 500 µm; (B, F, I–K) 50 µm; (C, D, G) 100 µm; (E) 1000 µm; (H) 200 µm. G and I not labeled.

In the normal mouse lung, the bronchiolar epithelia are covered by Clara and ciliated cells, whereas the alveolar epithelia are covered by AT1s interspersed with AT2s. We consistently observed that staining of the Clara cell marker secretoglobin family 1A member 1 [Scgb1a1 or Clara cell secretory protein (CCSP)] was confined to bronchiolar epithelia and that staining of the AT2 cell marker pro-surfactant protein C (pro-SPC) was dispersed among AT1s that were positive for podoplanin (PDPN) (Figure 1A, B). Lymphatic endothelial cells also express PDPN; because there is no lymphatics in alveolar area [22] PDPN can serve as a specific marker for AT1s. Bleomycin-induced alveolar epithelial damage was observed as early as 3 days post treatment. By 7 days post-treatment, large areas of alveolar epithelia lost both PDPN and pro-SPC staining, while infiltrating cells were evident as indicated by dense DAPI staining of nuclei (Figure 1C). Influenza virus infection caused damages of both bronchiolar and alveolar epithelia as early as 3 days post infection (dpi). Severe infiltration in alveolar area and loss of AT2s and AT1s were evident starting 5 dpi (Figure 1D).

Mice treated with bleomycin were visibly sick by 7 days post-treatment; with some of them showing severe clinical signs after 14 days and had to be euthanized. By 21 days post-treatment, the remaining mice appeared to have recovered from bleomycin treatment, and immunofluorescent analysis of lung sections at day 21 revealed a reduced cell infiltration in the lungs. Strikingly, large numbers of pro-SPC-positive AT2s were detected in clusters in the damaged parenchyma (Figure 1E, F), consistent with previous studies [12], [13], [15]. Similarly, in influenza virus-infected mice, newly generated AT2s appeared in the damaged parenchyma starting around 11 dpi, and large numbers of these cells were observed by 17 dpi (Figure 1H). Importantly, PDPN-positive cells with AT1 morphology were also detected among the newly generated AT2s (Figure 1G, I), indicating the repair of the damaged alveolar epithelia. These results suggest that both bleomycin treatment and influenza virus infection induce alveolar epithelial damages which are repaired during recovery.

To investigate the origin of the newly generated AT2s in the damaged parenchyma, we assayed proliferation of AT2s and Clara cells by BrdU labeling. Mice were infected with influenza virus and every two days between day 3 and 21 post infection, a group of mice were given BrdU for 2 hrs before analysis. Only a small fraction of AT2s incorporated BrdU during the entire course of the repair process. Quantification of representative BrdU stains at 19 dpi showed only 2.6±1.1% (mean±SE, 3 mice) of AT2s in the damaged parenchyma were labeled with BrdU and the labeled cells were scattered among non-labeled AT2s (Figure 1J). In contrast, a significant level of Clara cells was labeled with BrdU starting 5 dpi and at the peak of BrdU incorporation (9 dpi) 32.7±1.1% (mean±SE, 3 mice) of Clara cells in the damaged bronchioles were BrdU positive (Figure 1K). These results suggest that most of the new AT2s may not be generated through proliferation of pre-existing AT2s, consistent with the recent report [15].

To investigate the role of Clara cells in the regeneration of alveolar epithelia, we crossed Scgb1a1-CreER gene targeted mice [7] with ACTB-mT-EGFP transgenic mice to generate Scgb1a1-CreER:ACTB-mT-EGFP double transgenic mice. In this system, a floxed tomato red (mT) cassette driven by the beta-actin promoter (ACTB) is expressed in all cell types and tissues [23]. CreER recombinase under the control of the endogenous mouse Scgb1a1 promoter is expressed in Scgb1a1-expressing cells but retained in the cytoplasm. Following tamoxifen (TMX) treatment of the mice, CreER is translocated into the nucleus where it mediates recombination to activate the expression of EGFP by deleting the mT cassette. Thus, Scgb1a1-expressing cells and their progeny are labeled with EGFP following TMX treatment. EGFP and mT signals can be directly detected from tissue sections after formalin fixation and paraffin embedding.

**Figure 2:**
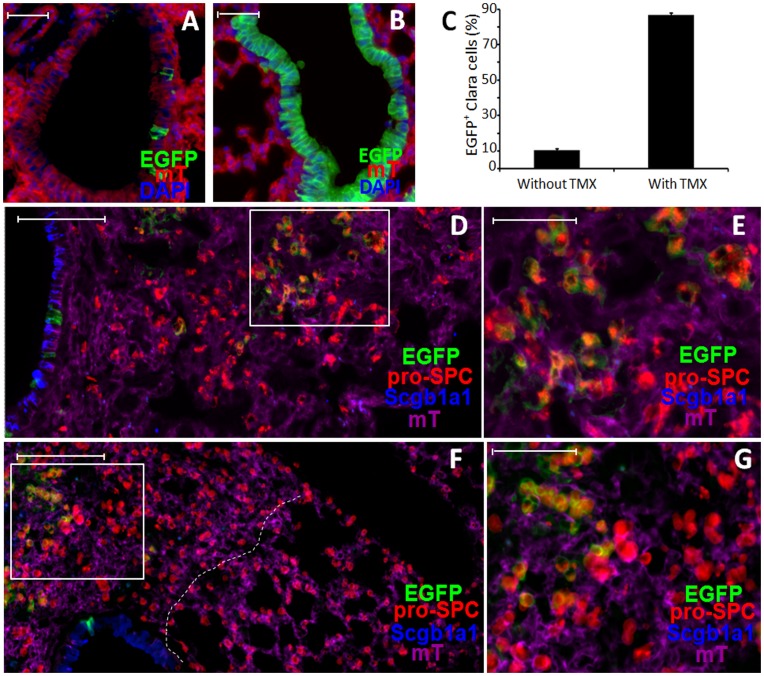
Induction of EGFP-positive AT2s in transgenic mice without TMX treatment. **A&B.** Representative images of bronchioles of Scgb1a1-CreER:ACTB-mT-EGFP transgenic mice without (A) or with (B) TMX treatment. Expression of EGFP (green) and tomato red (mT, red) are depicted. Sections were counterstained with DAPI (blue). **C.** Percentages (mean ± S.E.) of Clara cells per bronchiole that expressed EGFP in Scgb1a1-CreER:ACTB-mT-EGFP transgenic mice without or with TMX treatment. **D–G.** Scgb1a1-CreER:ACTB-mT-EGFP transgenic mice without TMX treatment were treated with bleomycin (D) or infected with influenza virus (F). Shown are representative images of lung sections analyzed for expression of EGFP (green) and tomato red (purple), and stained for pro-SPC (red) and Scgb1a1 (blue) at 21 days post-bleomycin treatment (D) or 17 days post-infection (F). Higher magnification images of boxed areas in (D) and (F) are shown as (E) and (G), respectively. White broken line in (F) demarcates the infiltrated area (left) from the normal area (right) of the lung. Scale bars: (A, B, D, F) 100 µm; (E, G) 50 µm.

To perform lineage tracing, we compared induction of EGFP-positive cells in double-transgenic mice in the presence or absence of TMX. In untreated double transgenic mice (without TMX), no EGFP-positive cells were observed in the alveolar region, and only ∼10% of Clara cells in the bronchioles were EGFP-positive (Figure 2A, C). Following TMX treatment, approximately 85% of Clara cells in the bronchioles were EGFP-positive (Figure 2B, C), and ∼7% of AT2s were also EGFP-positive. These results are consistent with those reported previously [7]. Surprisingly, we found that in the double transgenic mice even without TMX treatment, a small fraction of AT2s in the damaged lung regions were also labeled with EGFP during repair following either bleomycin treatment or influenza virus infection (Figure 2D–G). Since no alveolar cell was labeled by EGFP without injury and without TMX treatment, the induction of EGFP-positive AT2s following pulmonary damage in the absence of TMX treatment raises the possibility that these cells are derived from pre-labeled EGFP-positive Clara cells.

**Figure 3:**
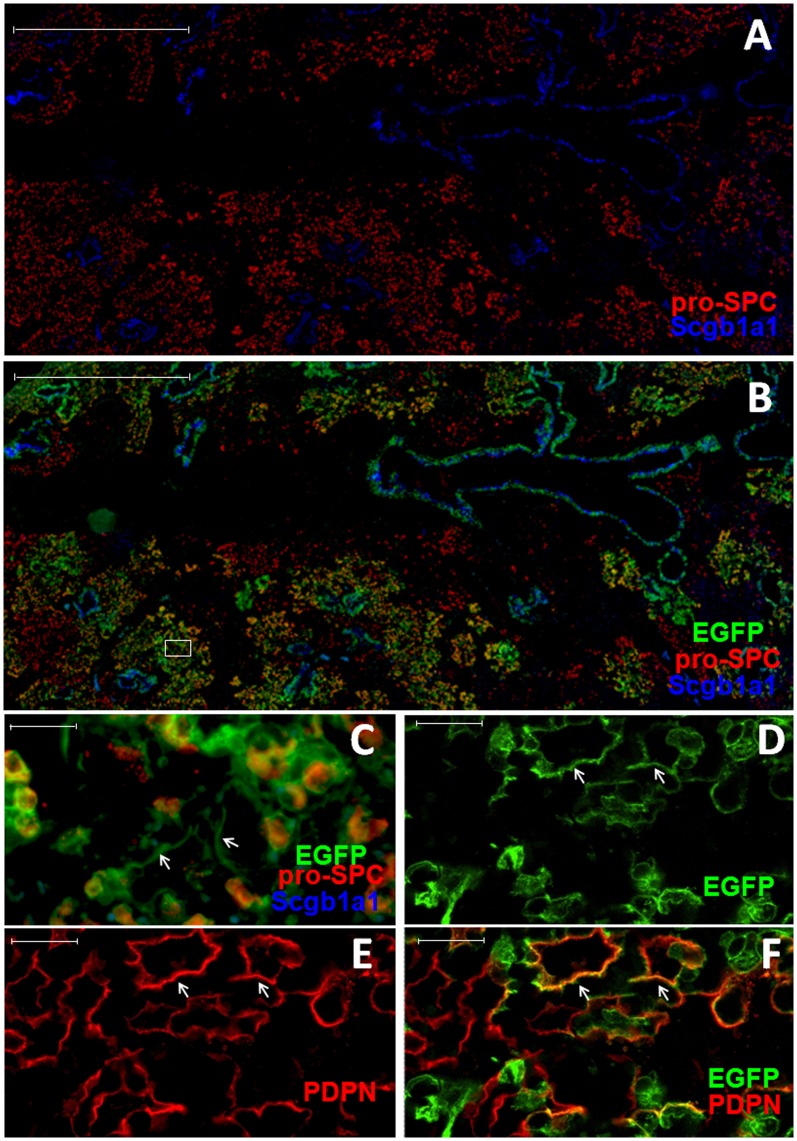
Observation of EGFP-positive AT2s and AT1s in TMX-treated transgenic mice after bleomycin treatment. **A–C.** Scgb1a1-CreER:ACTB-mT-EGFP transgenic mice were given tamoxifen and then treated with bleomycin. Shown are representative images of lung sections analyzed for EGFP (green), pro-SPC (red) and Scgb1a1 (blue) at 21 days post-treatment. Tomato red is not shown. Higher magnification of boxed area in (B) is shown as (C). Arrows in (C) indicate EGFP-positive AT1-like cells. **D–F.** Representative images of confocal analysis for EGFP (D), PDPN (E) and merged (F) of lung sectons from TMX-treated transgenic mice at 21 days post-bleomycin treatment. Arrows in (D–F) indicate EGFP and PDPN double-positive AT1s. Scale bars: (A, B) 1000 µm; (C–F) 20 µm.

**Figure 4:**
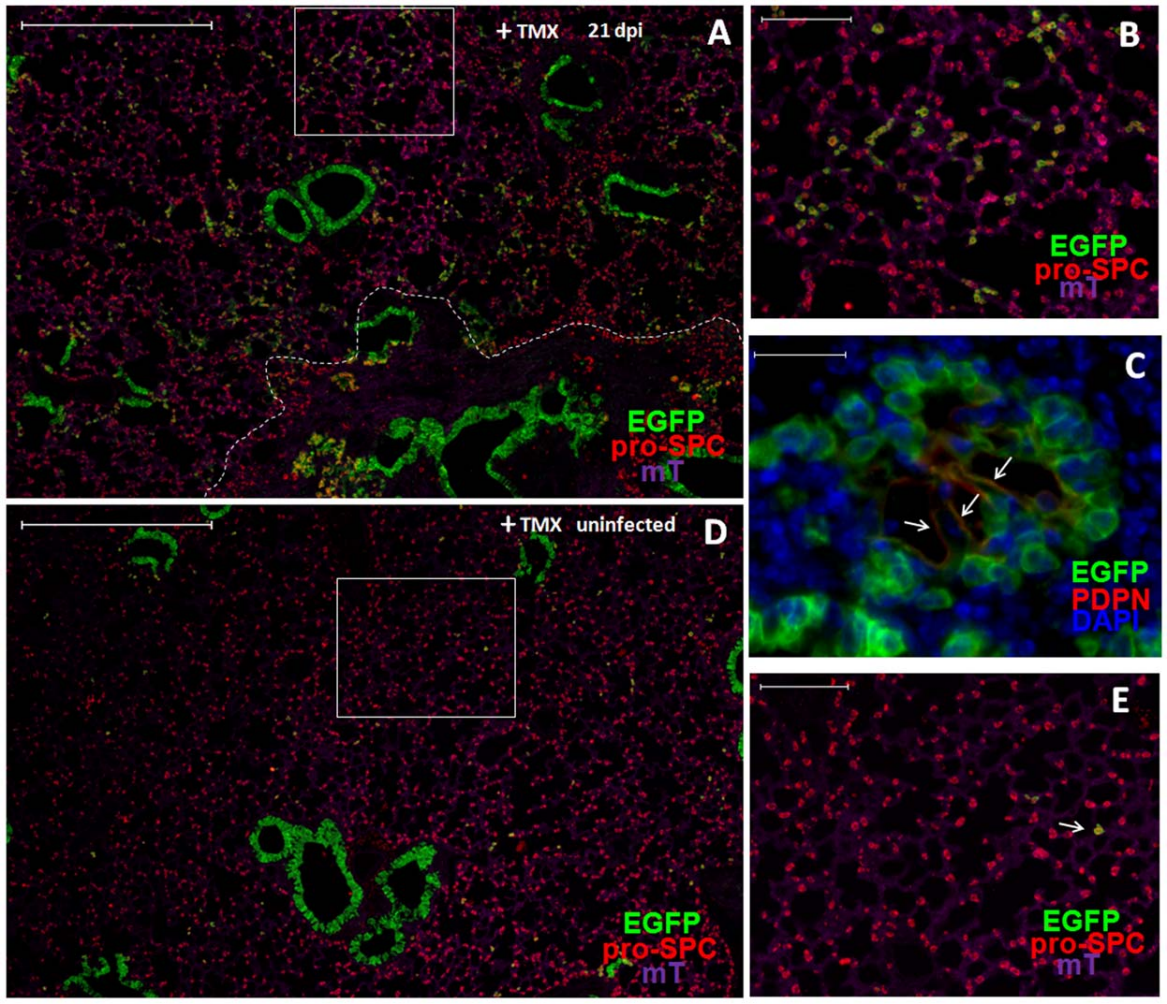
Observation of EGFP-positive AT2s and AT1s in TMX-treated transgenic mice after influenza virus infection. A–E. Representative images of lung sections of TMX-treated Scgb1a1-CreER:ACTB-mT-EGFP transgenic mice after (at 21 dpi) (A–C) and before (D–E) influenza infection. Expression of EGFP (green), tomato red (purple) and pro-SPC (red) are analyzed in (A, B, D, E). Higher magnification images of selected areas in (A) and (D) are shown as (B) and (E), respectively. White broken line in (A) demarcates the infiltrated area (below) and normal area (above). Expression of EGFP (green) and PDPN (red) with DAPI (blue) staining are analyzed in (C). Arrows in (E) indicate EGFP and PDPN double-positive cells. Scale bars: (A, D) 500 µm; (B, E) 100 µm; (C) 20 µm.

To further investigate this possibility, we analyzed induction of EGFP-positive cells in double-transgenic mice following lung injury in the presence of TMX. Transgenic mice were given TMX first to induce EGFP expression in Scgb1a1-positive cells and then treated with bleomycin or infected with influenza virus. Twenty-one days post bleomycin treatment, large numbers of pro-SPC-positive AT2s were observed to express EGFP throughout the damaged regions (Figure 3A, B). Quantification of five consecutive lung sections spanning more than 600 µm thickness showed that 77.9±1.6% (mean±SE) of AT2s were EGFP-positive. Among the EGFP-positive AT2s, many AT1-like cells were also EGFP-positive (Figure 3C, arrows). Confocal analysis of PDPN staining of adjacent lung sections confirmed the presence of EGFP-positive AT1 cells in the same region (Figure 3D–F, arrows). Similarly, following influenza virus infection of TMX-treated transgenic mice, EGFP-positive AT2s were also observed throughout the newly regenerated alveolar regions (Figure 4A, B). Quantification of EGFP-positive cells showed that 48.1±3.0% (mean±SE) of the AT2s in the damaged parenchyma were positive for EGFP. Furthermore, the EGFP-positive AT1s were also detected among EGFP-positive AT2 cells (Figure 4C). In comparison, only very few EGFP-positive AT2s were observed in the alveoli of non-infected mice (Figure 4D, E). These results show that Scgb1a1-expressing cells are the major cell types that give rise to the newly generated AT1s and AT2s.

## Discussion

In this study, we investigated regeneration of alveolar epithelia following severe lung damage induced by a small molecular weight chemical (bleomycin) or a natural infectious agent (influenza virus). Using lineage tracing, we provide definitive evidence showing that Scgb1a1-expressing cells can differentiate into AT2s and AT1s to repair the alveolar damage. After severe pulmonary damage induced by either bleomycin treatment or influenza virus infection, large proportions of newly generated AT2s and AT1s were labeled with EGFP in Scgb1a1-CreER:ACTB-mT-EGFP double transgenic mice (Figure 3 and 4). While the present study was prepared for publication, using tomato red as the genetic marker, Rock et al reported the observation of tomato red-labeled AT2s and AT1s in a similar lineage tracing system following bleomycin treatment [14]. Compared to their study, which only used bleomycin and did not quantify the percentage of labeled AT2s, we performed a more quantitative analysis and also used a natural infectious agent influenza virus besides bleomycin. Together, these findings unequivocally demonstrate that labeled AT2s and AT1s are derived from pre-labeled Scgb1a1-expressing cells.

In the Scgb1a1-CreER system, Cre-mediated recombination labels predominantly Clara cells, some AT2s, BASCs and occasionally ciliated cells [7] and therefore the EGFP-positive AT1s and AT2s could originate from one of the four cell types. Although the putative BASCs can differentiate into both Clara cells and AT2s *in vitro* [16], they are too rare [16]–[18] to contribute significantly to the newly generated EGFP-positive AT2s and AT1s. Similarly, only ∼0.4% of ciliated cells were labeled by EGFP with or without TMX treatment [7]. They are unlikely to have contributed significantly to the large numbers of the EGFP-positive AT2s and AT1s following bleomycin treatment or influenza infection. Approximately 7% of AT2s were labeled by EGFP after TMX treatment without lung injury, raising the possibility that these pre-labeled AT2s may give rise to the newly generated EGFP-positive AT2s in the damaged parenchyma. However, this is unlikely a major pathway for the following reasons: First, after bleomycin treatment or influenza infection there were almost no AT2s left in the damaged parenchyma (Figure 1C, D). If TMX-induced EGFP-positive AT2s contributed to the regeneration of AT2s in the damaged parenchyma, they would have to come from un-damaged lung area. Currently, there is no evidence supporting this mechanism. Second, for the 7% of EGFP-positive AT2s to give rise to ∼80% of EGFP-positive AT2s following bleomycin treatment and 50% following influenza infection, the 7% EGFP-positive cells would have to expand disproportionally. However, only very few of AT2s were in proliferation throughout the 21 days of the repair process that was monitored in the present study (Figure 1J). Third, it is possible that lung injury may induce expression of Scgb1a1 in the newly generated AT2s. However, this alone should not induce more EGFP-positive AT2s because Cre-mediated recombination also requires TMX treatment. In our experimental system, TMX was given at least one week before infection or bleomycin treatment, whereas large numbers of new AT2s are not induced until two weeks after lung injury. Finally, a recent study using direct lineage tracing of AT2s in bleomycin-induced lung injury provides direct evidence showing that the majority of the newly generated AT2s are not derived from the pre-existing AT2s [15]. Therefore, the 7% pre-labeled AT2s in TMX treated mice are unlikely to give rise to the majority of EGFP-expressing AT2s during the alveolar regeneration following bleomycin treatment or influenza infection.

In contrast, 85% of Clara cells were labeled with EGFP following TMX induction and they underwent rapid proliferation following influenza induced damage of bronchiolar epithelia (Figure 1K). There are sufficient numbers of EGFP-labeled Clara cells that could give rise to a large number of AT2s and AT1s in the damage parenchyma. In our lineage tracing system, not all the Clara cells were labeled with EGFP, and hence not all the newly generated AT2s were labeled. Furthermore, we show that when most Clara cells (∼85%) were labeled by EGFP after TMX treatment (without lung injury), ∼7% of AT2s were also positive for EGFP. These EGFP-positive AT2s could be generated because of weak expression of Scgb1a1 in a small portion of AT2s [7]. However, if Scgb1a1 transcript was restricted to the conducting airways in mice [24], it is more likely that some EGFP-labeled Clara cells naturally differentiate into AT2s in the absence of lung injury. The fact that most of the EGFP-labeled AT2s were close to BADJs strongly supports the latter possibility [7]. In addition, by over-expression of fibroblast growth factor 10 (FGF10) or activation of the Wnt/β-catenin signaling in combination with expression of a mutant kras, two recent studies showed that Clara cells can be induced to express pro-SPC and become stem cell/progenitor-like cells [25], [26], indicating the progenitor potential of Clara cells for alveolar regeneration. Thus, although we could not exclude completely the contribution of BASCs, ciliated cells, existing AT2s or other progenitor cells to give rise to AT2s and AT1s during the regeneration of new alveolar epithelia, our findings support that a major fraction of the newly generated AT2s are likely derived from Clara cells.

Our findings provide a possible explanation for other putative stem and progenitor cells that were reported to give rise to AT2s. For example, a recent study showed that α6β4-positive cells isolated from adult mouse lung can give rise to Clara cells and AT2s in kidney capsules when mixed together with fetal lung cells. Since ∼10% of the purified α6β4-positive cells were strongly positive for Scgb1a1 [15], these pre-existing Clara cells may have given rise to Clara cells and AT2s in the engrafted renal capsule. More recently, p63^+^Krt14^+^Krt5^+^PDPN^+^ cells were observed in the damaged lung parenchyma following influenza virus infection and proposed to contribute to alveolar epithelial regeneration [20]. Even though these cells can be labeled in Krt14-CreER transgenic system, stringent lineage tracing has yet to be performed. In our study, following either bleomycin treatment or influenza virus infection, PDPN-positive AT1s re-appeared in the damaged parenchyma where there were large numbers of newly generated AT2s. Such areas were no longer severely infiltrated, clearly indicating the regeneration of damaged alveolar epithelia (Figure 1), which is congruent with previous reports [12], [13], [15].

Our study reveals an underappreciated cellular mechanism where Clara cells serve as the progenitors for alveolar epithelial regeneration during severe lung damage repair and perhaps during normal homeostasis. Although an early study suggested two mechanisms, i.e. in damaged areas away from bronchioles, alveolar epithelium is repaired via proliferation of existing AT2s; whereas in areas near bronchioles, progenitor cells from bronchiolar epithelia may differentiate into AT2s to regenerate alveolar epithelia [13]. For a long time, it is believed that the pre-existing AT2s play the major role in alveolar epithelial regeneration. Using a genetic lineage tracing system to specifically label AT2s, a recent study showed that the newly generated AT2s are not from pre-existing AT2s, indicating that other progenitor cells give rise to new AT2s for the repair of alveolar epithelia [15]. By genetically tracing Scgb1a1-expressing cells, our study provides most direct evidence that Clara cells from bronchiolar epithelia likely play a major role in alveolar epithelial regeneration following severe pulmonary damage whether chemically or virally induced. Future investigations on the cellular and molecular mechanisms of this differentiation process will facilitate the search for novel therapeutic strategies for severe pulmonary injury.

## Materials and Methods

### Mice, Influenza Virus Infection, and Chemical Treatment

C57BL/6 (B6) mice were purchased from the Center for Animal Resources, Singapore. Transgenic ACTB-mT-EGFP (stock number 007676) mice on the B6 background were purchased from The Jackson Laboratories. Scgb1a1-CreER knockin mice (MGI) on the B6 background were kindly provided by Dr. Brigid Hogan of Duke University, USA. Double Scgb1a1-CreER:ACTB-mT-EGFP transgenic mice were generated by mating Scgb1a1-CreER mice with ACTB-mT-EGFP mice, and progeny were screened according to the protocol provided by Dr. Hogan’s laboratory. To induce Cre-mediated recombination, Scgb1a1-CreER:ACTB-mT-EGFP transgenic mice were treated with tamoxifen in corn oil (or corn oil only as control) every other day for four times at a dose of 0.25 mg/g body weight [7]. One week after the last TMX injection (or two weeks after the initiation of TMX treatment) mice were infected with influenza virus A/Puerto Rico/8/34 H1N1 (PR8). B6 and transgenic mice aged 8–12 weeks old were challenged with a sublethal dose of 100 pfu of PR8 virus per mouse, or treated with bleomycin (0.5–1 U/kg body weight) by intratracheal instillation under anesthesia. BrdU solution (BD Pharmingen) was injected intraperitoneally 2 hours before sacrifice at a dose of 50 mg/kg body weight. All animals were housed and handled in biosafety level 2 animal facilities. All animal protocols were approved by the Institutional Animal Care and Use Committees of National University of Singapore and Massachusetts Institute of Technology.

### Antibodies for Immunochemistry

Polyclonal rabbit anti-Scgb1a1 antibody (US Biological, C5828) was used at 1∶200 dilution. Polyclonal goat anti-pro-SPC (Santa Cruz Biotechnology, sc-7706), goat anti-PDPN (R&D Systems, AF3244), rabbit anti-GFP (Abcam, ab290) and mouse anti-BrdU (Leica Microsystems, NCL-BrdU) were used at a 1∶50 dilution. Secondary antibodies (including donkey anti-rabbit, anti-goat, anti-rat, or anti-mouse), each with different Alexa Fluor conjugations, were all purchased from Life Technologies and used at 1∶200 dilution.

#### Histopathology and immunohistochemical staining

Mouse lung tissues were harvested and fixed in 10% neutral buffered formalin solution (Sigma-Aldrich) for 24 h. The tissues were processed with a tissue processor (Leica Microsystems) and embedded in paraffin. Sections were cut at 5 µm thickness, mounted on polylysine-coated slides (Thermo Fisher Scientific), dewaxed, and rehydrated. For immunofluorescent staining, antigen retrieval was carried out by digestion in proteinase K solution (Sigma-Aldrich, 20 ug/ml, in 50 mM Tris-Cl, 1 mM EDTA, pH 8.0) at 37°C for 20 min. For BrdU staining, sections were processed according to the manufacturer’s protocol. Sections were then immersed for 1 h in blocking buffer (3% BSA, 0.2% Triton X-100 in PBS), incubated with primary antibody (in blocking buffer) at 4°C overnight, followed by incubation with secondary antibody at 4°C for 1 h. Sections were mounted with anti-fade reagent (Life Technologies), scanned with a high-resolution MIRAX MIDI system (Carl Zeiss) equipped with both bright field and fluorescence illumination. Images were analyzed using the Mirax viewer software. Confocal microscopy was performed using FluoView FV1000 (Olympus).
